# PHENOPSIS DB: an Information System for *Arabidopsis thaliana *phenotypic data in an environmental context

**DOI:** 10.1186/1471-2229-11-77

**Published:** 2011-05-09

**Authors:** Juliette Fabre, Myriam Dauzat, Vincent Nègre, Nathalie Wuyts, Anne Tireau, Emilie Gennari, Pascal Neveu, Sébastien Tisné, Catherine Massonnet, Irène Hummel, Christine Granier

**Affiliations:** 1 Laboratoire d'Ecophysiologie des Plantes sous Stress Environnementaux (LEPSE), INRA-AGRO-M, UMR 759, 2 Place Viala, 34060 Montpellier Cedex 1 France; 2 Mathématiques, Informatique et Statistique pour l'Environnement et l'Agronomie (MISTEA), INRA-AGRO-M, UMR 729, 2 Place Viala, 34060 Montpellier Cedex 1 France; 3 Institut Jean-Pierre Bourgin, UMR1318 INRA-AgroParisTech, Versailles, France; 4 Ecologie et Ecophysiologie Forestières INRA, Nancy Université, UMR1137, IFR 110 EFABA, F-54280 Champenoux, France

## Abstract

**Background:**

Renewed interest in plant × environment interactions has risen in the post-genomic era. In this context, high-throughput phenotyping platforms have been developed to create reproducible environmental scenarios in which the phenotypic responses of multiple genotypes can be analysed in a reproducible way. These platforms benefit hugely from the development of suitable databases for storage, sharing and analysis of the large amount of data collected. In the model plant *Arabidopsis thaliana*, most databases available to the scientific community contain data related to genetic and molecular biology and are characterised by an inadequacy in the description of plant developmental stages and experimental metadata such as environmental conditions. Our goal was to develop a comprehensive information system for sharing of the data collected in PHENOPSIS, an automated platform for *Arabidopsis thaliana *phenotyping, with the scientific community.

**Description:**

PHENOPSIS DB is a publicly available (URL: http://bioweb.supagro.inra.fr/phenopsis/) information system developed for storage, browsing and sharing of online data generated by the PHENOPSIS platform and offline data collected by experimenters and experimental metadata. It provides modules coupled to a Web interface for (i) the visualisation of environmental data of an experiment, (ii) the visualisation and statistical analysis of phenotypic data, and (iii) the analysis of *Arabidopsis thaliana *plant images.

**Conclusions:**

Firstly, data stored in the PHENOPSIS DB are of interest to the *Arabidopsis thaliana *community, particularly in allowing phenotypic meta-analyses directly linked to environmental conditions on which publications are still scarce. Secondly, data or image analysis modules can be downloaded from the Web interface for direct usage or as the basis for modifications according to new requirements. Finally, the structure of PHENOPSIS DB provides a useful template for the development of other similar databases related to genotype × environment interactions.

## Background

*Arabidopsis thaliana*, a small flowering plant with a rapid life cycle, offers important advantages for researches in genetics and molecular biology. Since 2000, the complete sequencing of its genome has enabled scientists to monitor gene expression on a genome-scale [1] in different organs and in different environmental conditions [e.g. [2,3]]. The broad-based knowledge of this plant includes extensive genetic maps of all five chromosomes, efficient technology for mutagenesis and transformation and a large range of biological resources available at the various Arabidopsis stock centers (Arabidopsis Biological Resource Center, Nottingham Arabidopsis Stock Center, Riken Bioresource Center, INRA-Versailles Genomic Resource Center and Lehle Seeds, a private company). Many structured databases and querying tools have been developed providing repositories of large datasets and efficient applications for the determination of gene function (TAIR [4], NASC Proteomics [5], etc). While these databases provide extensive and robust genetic or molecular information, metadata like the precise characterisation of environmental conditions or plant developmental phenotypes are generally poorly documented. This point has recently received attention and several guidelines have been proposed acknowledging the importance of comprehensive metadata, and thus allowing cross-validation of experiments and meta-analysis procedures [6-10].

Unravelling gene function by large scale mutant screening has been mainly based on the mean value of a phenotypic effect measured under a given lab condition. It is often assumed in this approach that phenotypic variation among plants is largely due to genotypic variation. However, the validity of this assumption was questioned by a recent study in which three genotypes of *Arabidopsis thaliana *were grown in 10 laboratories using the same standardised conditions [11]. Despite the use of a common, highly detailed protocol, the 10 labs still obtained phenotypic variation within genotypes for molecular and leaf developmental traits. The results showed that even small differences in environmental conditions or plant handling substantially affected growth at different levels [11]. This study clearly demonstrates the need for precise recording of environmental conditions and reproducible characterisation of phenotypic traits in order to enable data sharing and comparison across laboratories. While automated phenotyping platforms are developed in many groups to obtain precise records of plant environmental conditions and growth phenotypes (Traitmill [12], PHENOSCOPE [13], WIWAM [14]), these data are still not available through repository databases. One of the pioneer platforms for reproducible phenotyping of *Arabidopsis thaliana *was the PHENOPSIS platform developed in our group in 2003 [15]. In three highly controlled growth chambers, plants are subjected to different temperatures, day-lengths and drought treatments with an automatic recording of all environmental data. In platforms such as this, large quantities of environmental data, plant images and phenotypic data are produced for the study of genotype × environment effects on different plant processes. Procedures need to be conceived for a proper handling of these datasets, their efficient extraction and sharing with the scientific community. Here, we describe the content and utility of PHENOPSIS DB, an information system for the storage (database), analysis and sharing (Web interface, Web Services) of images and data collected in the PHENOPSIS platform.

## Construction and content

### Data source

PHENOPSIS DB contains phenotypic data and experimental and environmental metadata (see additional file 1: Description of the variables stored in PHENOPSIS DB). The phenotypic data include online (*i.e. *automatically recorded) and offline (*i.e. *manually recorded) plant images and sets of offline phenotypic measurements. Metadata consist of protocols, descriptions of variables, genotype characteristics and online environmental data.

#### Experiment protocols and variable descriptions

Each experiment is associated with a protocol that gives information about the experimental context. Other protocols describe how variables were obtained to ensure that all experimenters use the same methods to measure a given variable.

#### Genotype characteristics

*Arabidopsis thaliana *genotypes may include ecotypes, inbred lines from specific crosses, mutants, etc. and information on the specific features of the genotype and the source of the material, i.e. the laboratory or stock center providing the seeds.

#### Environmental conditions

Climatic conditions (air temperature, air humidity, light intensity, vapor pressure deficit) in the PHENOPSIS growth chambers are continuously recorded during an experiment [15] and automatically sent to the server. R [16] functions check and insert them into the database. Plant watering data, *i.e. *the weight of individual pots before and after watering and the supplied amount of nutrient solution [15], are also automatically recorded and inserted into the database via real-time automated SQL requests.

#### Images

**Figure 1:**
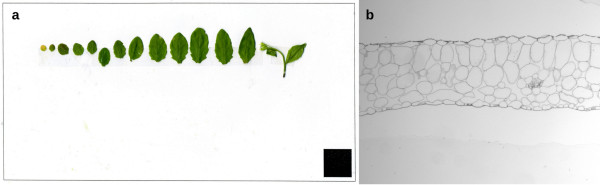
**Examples of images produced by experimenters**. (a) Scan of individual rosette leaves of an *Arabidopsis thaliana *plant allows to estimate leaf area using a macro developed in ImageJ [18]. (b) Histological section of an individual leaf of *Arabidopsis thaliana *allows to measure leaf thickness and the proportions of individual leaf tissues.

Visible and infrared images of each individual plant in PHENOPSIS [15] are automatically transferred in real-time to the server. Additional offline images are manually inserted into the database. These are produced by experimenters after the harvest of plants or plant organs for destructive measurements, including scans of different plant parts (roots, leaves, etc) (Figure 1a), or obtained after organ preparation and microscopic observations (Figure 1b).

#### Phenotypic data measured on plants

Non-invasive measurements, such as rosette and individual leaf area determination, plant growth stage records and transpiration measurements are performed during a growth run within PHENOPSIS. Invasive measurements, on the other hand, require the harvest of plants or plant parts and are performed at predefined dates (x days after sowing) or at given plant developmental stages. Examples are the determination of plant and organ fresh and dry weight, leaf thickness, leaf epidermal cell density and stomatal density. Both invasive and non-invasive measurements are inserted into the database via the Web interface. R functions are used to check data consistency before insertion.

#### Data volume

Currently, 70 experiments are stored in the database and 15 of them are publicly available. They include 87000 phenotypic measurements on 865 genotypes, of which 50000 measurements on 620 genotypes are publicly available. 600000 images are stored in the database and more than 90000 are publicly available.

### PHENOPSIS DB information system

**Figure 2:**
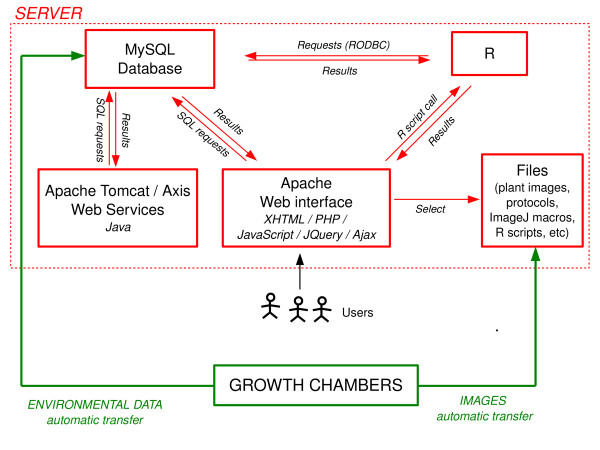
**Overview of the PHENOPSIS DB Information System**. Database, Web interface, Web Services, R functions and files (plant images, protocol files, etc) are stored on a Linux server. Environmental data from the growth chambers are automatically inserted into the database, and visible/infrared images are automatically stored and organized on the server. Users interact with the Web interface for offline data, metadata insertion, data consultation and analysis. The connection to the database is either directly performed with SQL requests, or indirectly via R scripts using the RODBC package for data formatting or analysis. Web Services connect to the database for automated data extraction.

The PHENOPSIS DB has been designed for data storage, browsing and retrieval. It also provides tools for data visualisation and analysis, and image analysis. It consists of three major components: the database, the Web interface with modules developed in R or ImageJ [17], and several Web Services (Figure 2).

#### The database

The database was developed using the MySQL 5.0 Community Server and is composed of 15 physical tables (see additional file 2: Description of the physical data model of the PHENOPSIS DB database).

#### The Web interface

The Web interface was developed using XHTML, PHP, JavaScript, Jquery, Ajax and CSS. Both CSS and XHTML scripts respect the W3C [18] standards and were validated by W3C online tools [19,20]. PHP scripts call R functions to check, insert and format data, and to perform online statistical analysis or visualisation. The RODBC package in R version 2.9.2 was used to establish the database connection.

#### User access

All metadata are freely available without restriction or authentication request. Metadata include: characteristics of experiments and associated protocols, list of genotypes grown in an experiment, list of variables measured in an experiment with their definition and associated protocols, comments on the experiments, micrometeorological data and plant watering data.

Images and phenotypic data from public experiments and public genotypes are also freely available without restriction or authentication request. The whole dataset associated with an experiment and/or a genotype becomes public as soon as the data have been published.

The access to images and phenotypic data from non-published experiments or confidential genotypes requires a user authentication that can be requested from the administrator in charge of the information system.

#### Web Services

Web Services were developed to enhance interoperability and data exchanges with other systems (information systems, stand-alone programs). The PHENOPSIS DB Web Services are based on the Tomcat/Axis solution, described using WSDL language and they apply the SOAP protocol. They were developed in the Java language.

## Utility and discussion

### PHENOPSIS DB Web interface

#### A user-friendly Web interface

Centralised information systems are often developed for data storage when datasets are too extensive for personal computers. They are also used to promote exchanges between researchers and to perform meta-analyses, requiring high traceability and reproducibility of datasets. This can only be ensured through comprehensive metadata, data collection protocols and data descriptions. The PHENOPSIS DB interface has been developed for a large scientific community and allows the browsing, downloading, visualisation and analysis of all data recorded in the PHENOPSIS platform. The PHENOPSIS platform and the information system structure are documented on the Web interface (see http://bioweb.supagro.inra.fr/phenopsis/Accueil.php?lang=En). In the *Data Browsing and Download *section, basic or advanced searches can be performed depending on the user's familiarity with the system.

#### Interoperability between PHENOPSIS DB and other databases

Both the use of standards and the integration of ontologies enhance the interoperability between PHENOPSIS DB and other biological databases. The genotype nomenclature is based on the TAIR international nomenclature [21,22] and hyperlinks lead to their description on the TAIR or NASC websites. The characterisation of growth stages follows the standard nomenclature described in [23]. Whenever possible, measured organs are characterised according to the plant structure proposed in Plant Ontology [6]. In addition, correspondence between plant growth variables and the ontologies of phenotypic traits were made. Some matches to variables were identified as terms in Trait Ontology [24], while for others it was necessary to combine different ontologies (Phenotype, Attribute and Trait Ontology [25], Plant Ontology, etc) following the EQV (Entity Qualifier Value) model [26]. Variables not clearly identified in existing ontologies were defined as precisely as possible and will be submitted to ontology consortiums.

#### Consultation of the experiments and/or genotypes

The *Experiments *subsection within the *Data Browsing and Download *section allows searches on experiments associated with a publication, given genotypes or a specific type of stress (see http://bioweb.supagro.inra.fr/phenopsis/ConsulterManip.php, e.g. select experiments without any environmental stress). In the advanced search, users can select additional filters such as measured variables, environmental conditions, etc. Each experiment is associated with a description that provides its general features, the genotypes studied and the variables measured, the characteristics of each pot (sowing date, weights for soil humidity calculation, etc), and the parameters for setting environmental conditions.

#### Download and analysis of phenotypic data

Users of the system can download the publicly available datasets in the *Data Browsing and Download > Data measured on plants *section (see http://bioweb.supagro.inra.fr/phenopsis/ConsulterMesurePlante.php), using similar searching criteria to those described above to restrict the downloading to specific data of interest.

**Figure 3:**
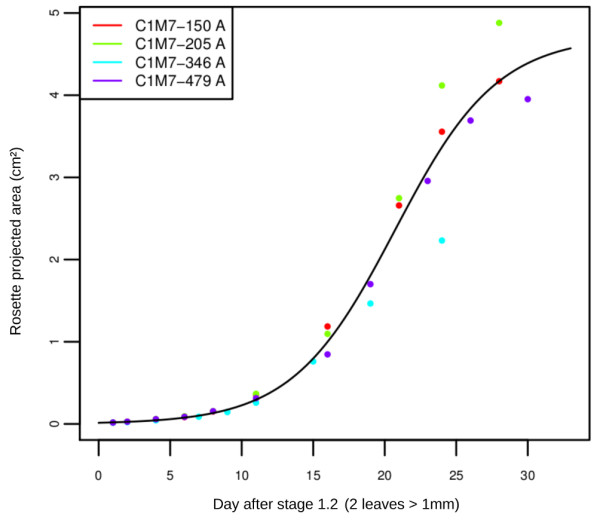
**Example of an online statistical analysis**. Projected rosette areas are plotted over time for four plants of the genotype LAF11-1 grown in four different pots (C1M7-150, C1M7-250, C1M7-346 and C1M7-479) in a same experiment (C1M7). A sigmoïdal model is fitted to the data. Projected rosette areas were obtained by the analysis of images taken by the automatons. This graph was produced on the PHENOPSIS DB Web interface in the *Graphs and Descriptive Statistics > Data measured on plants *section by selecting the experiment C1M7, the genotype LAF11-1, the phenotypic measure 'Rosette projected area', the sigmoïdal curve fitting analysis and the genotype level for the analysis.

Applications have been developed that assist users in the visualisation and statistical analysis of phenotypic data. They can be found in the *Graphs and Descriptive Statistics > Data measured on plants *section (see http://bioweb.supagro.inra.fr/phenopsis/StatPlante.php). Users can perform online univariate analyses, including histograms, boxplots or curve fitting related to growth kinetics (Figure 3). In addition, R scripts developed for specific analyses are available: sigmoidal curve fitting to leaf or cell expansion data, test of loci effects on quantitative variable correlations, and selection of Recombinant Inbred Lines. The R sources can be downloaded with their descriptions, test datasets and the corresponding outputs.

#### Download and visualisation of environmental conditions during an experiment

Environmental data, including micrometeorological and plant watering data, can be consulted and downloaded in the *Data Browsing and Download *section. Two modules have been developed in the *Graphs and Descriptive Statistics *section to check the consistency between set and obtained environmental conditions and to assist in the precise monitoring of experiments. In the first module, micrometeorological data and a basic statistical analysis can be visualised and downloaded in graphs. More specifically, the module displays the kinetics of the different meteorological data over an experiment together with a statistical summary (see http://bioweb.supagro.inra.fr/phenopsis/StatMeteo.php). In the second module, the soil water content in pots can be visualised and downloaded in graphs together with a basic statistical analysis (see http://bioweb.supagro.inra.fr/phenopsis/StatIrrigation.php). One application within the module displays the changes in soil humidity over an experiment for individual pots [15] with a statistical summary. A second application produces graphs showing the soil water content of all pots in a PHENOPSIS growth chamber before and after watering at a given date and for each plant watering cycle.

#### Download and analysis of images

Users of the system can download the publicly available images in the *Data Browsing and Download > Plant images *section (see http://bioweb.supagro.inra.fr/phenopsis/ConsulterImages.php) and can restrict the downloading by applying filters. Plant images can be previewed, downloaded in ZIP files and used in the estimation of additional variables by applying other image analysis algorithms. For example, scans that have been used for the measurement of individual area of successive leaves on a rosette can be re-analysed to estimate shape parameters of the same leaves; similarly, leaf sections that have been used in the estimation of leaf thickness can be used in the measurement of vein diameter.

The *Image Analyses and ImageJ Macros *section provides tools for the analysis of large sets of plant images in an automatic or semi-automatic way using ImageJ macros (see http://bioweb.supagro.inra.fr/phenopsis/MacroImageJ.php). These macros can be downloaded and run as a stand-alone application for the analysis of (i) batches of rosette images to measure the projected rosette area of individual plants and (ii) leaf scans to measure individual leaf areas.

### PHENOPSIS DB Web Services

Our Web Services implement several methods. Currently, in the main methods one can get the list and description of (i) the public genotypes studied in all experiments or in a specific experiment, (ii) the measured phenotypic variables or (iii) the different types of images collected. Additionally, it is possible to get the sequence of visible images taken automatically in the growth chambers for plants of a specific genotype grown in a specific experiment. Using this last method one can for example automatically generate animated images of individual plant growth. Some examples of client applications available in different languages (Python, PHP) can be downloaded from the Web interface.

The Web services are described at http://bioweb.supagro.inra.fr/phenopsis/WebService.php and available to client programs via the WSDL document http://bioweb.supagro.inra.fr/phenopsis/wsdl.

### Examples of applications

The utility of PHENOPSIS DB for the analysis of large datasets has been demonstrated in recent studies. In a first example, the multi-scale analysis of leaf growth in 120 genotypes allowed the identification of robust emergent properties in the sub-cellular control of leaf development [27]. Secondly, the comparison of the leaf growth response of the same 120 genotypes, grown in limited soil water content, allowed the detection of genotypes that maintained leaf growth under drought [28].

### Examples of extensions

The whole system is flexible and easily upgradable to host new environmental or phenotypic variables and new types of images resulting from the evolution of research projects or the development of new protocols. For example, the creation of new environmental variables associated with mineral and abiotic stresses in soil is in progress. In addition, the development of a recent protocol for the 3D characterisation of leaf growth at the cellular level [29] has required the creation of new phenotypic variables. Finally, as the platform is also used in the production of highly characterised leaf material for molecular, biochemical or mineral content analyses, variables will be extended to metabolites contents, enzyme activities, transcript profiling, etc [11,30].

## Conclusions

PHENOPSIS DB provides the storage of millions of data and hundreds of Gb of images generated yearly in the PHENOPSIS platform. The information system contains useful resources for the scientific community working on genotype × environment interactions in *Arabidopsis thaliana*. Moreover, its structure serves as a template for other groups developing similar systems.

## Availability and requirements

PHENOPSIS DB is an open access database: http://bioweb.supagro.inra.fr/phenopsis/

It is referenced by APP (French Agency for Program Protection) under the INRA name and with number IDDN.FR.001.160017.000.R.P.2010.000.40000.

Metadata, images and phenotypic data from public experiments and public genotypes can be downloaded for further analyses. However, all analyses or figures produced using data accessed via PHENOPSIS DB must include a clear indication of sources such as: "This analysis is based upon data provided by PHENOPSIS DB", with citation of this paper. In the case of private data the acknowledgement must also include a statement such as "Permission to use these data was granted by <name, title and affiliation>".

Our group will service PHENOPSIS DB continuously and update it on a regular basis. Questions, comments and requests regarding this database should be sent to Vincent Negre at vincent.negre@supagro.inra.fr.

## Authors' contributions

JF designed and implemented the database and the Web interface, developed R modules for graphical and statistical analyses, provided support on the automatic transfer of data, integrated the ontologies and designed the Web Service application. MD is responsible for the functioning of the PHENOPSIS platform and provided support on the automatic transfer of data. VN provided support on the automatic transfer of data, the integration of ontologies and the Web Service interface. NW developed the modules for image analysis. AT and PN provided support on the design of the database, the Web interface and the Web Service application, and in the integration of ontologies. EG developed the Web Service application. ST, CM and IH have made their data publicly available. CG conceived the study, participated in its design and coordination. JF and CG wrote the manuscript with the support of all other authors. All authors have approved the final submitted version.

## Supplementary Material

Additional file 1 **Description of the variables stored in PHENOPSIS DB**. Four types of variables have been defined: variables provided by the automatons, environmental instructions given by experimenters, meteorological variables in the growth chambers and variables measured on plants by experimenters. Click here for file

Additional file 2 **Description of the physical data model of the PHENOPSIS DB database**. Four tables allow the management of user rights (*Group, User*, *SpecialUser *and *GroupUser *tables). They provide authorisation on data access and data insertion and restrict the access to specific experiments and/or genotypes listed with their characteristics in the *Experiment *and *Genotype *tables respectively. The growth chamber in which a particular experiment is performed, the characteristics of the pots in this experiment and the environmental instructions provided by experimenters are listed in the *Chamber, Pot *and *Instruction *tables respectively. Five other tables are related to the studied variables and the parts of the plants they are measured on. All studied variables are defined in the *Variable *table and the plant parts on which they are measured are defined in the *Organ *table. Micro-meteorological data are stored in the *MeteoMeasurement *table. Plant watering data and names and filename of the images collected by the automatons are stored in the *AutomatonMeasurement *table. Offline phenotypic data are stored in the *OrganMeasurement *table, as well as file names of plant images taken by experimenters. A last table named *Comment *allows the storage of all events and remarks associated with an experiment. Additional supplementary material is available on the PHENOPSIS DB Web interface: http://bioweb.supagro.inra.fr/phenopsis/. Click here for file
