# Supplementary figures and images for: PHENOPSIS DB: an Information System for Arabidopsis thaliana phenotypic data in an environmental context
Source: BMC Plant Biol. 2011 May 9;11:77. doi: 10.1186/1471-2229-11-77 (PMC3112076; doi:10.1186/1471-2229-11-77)

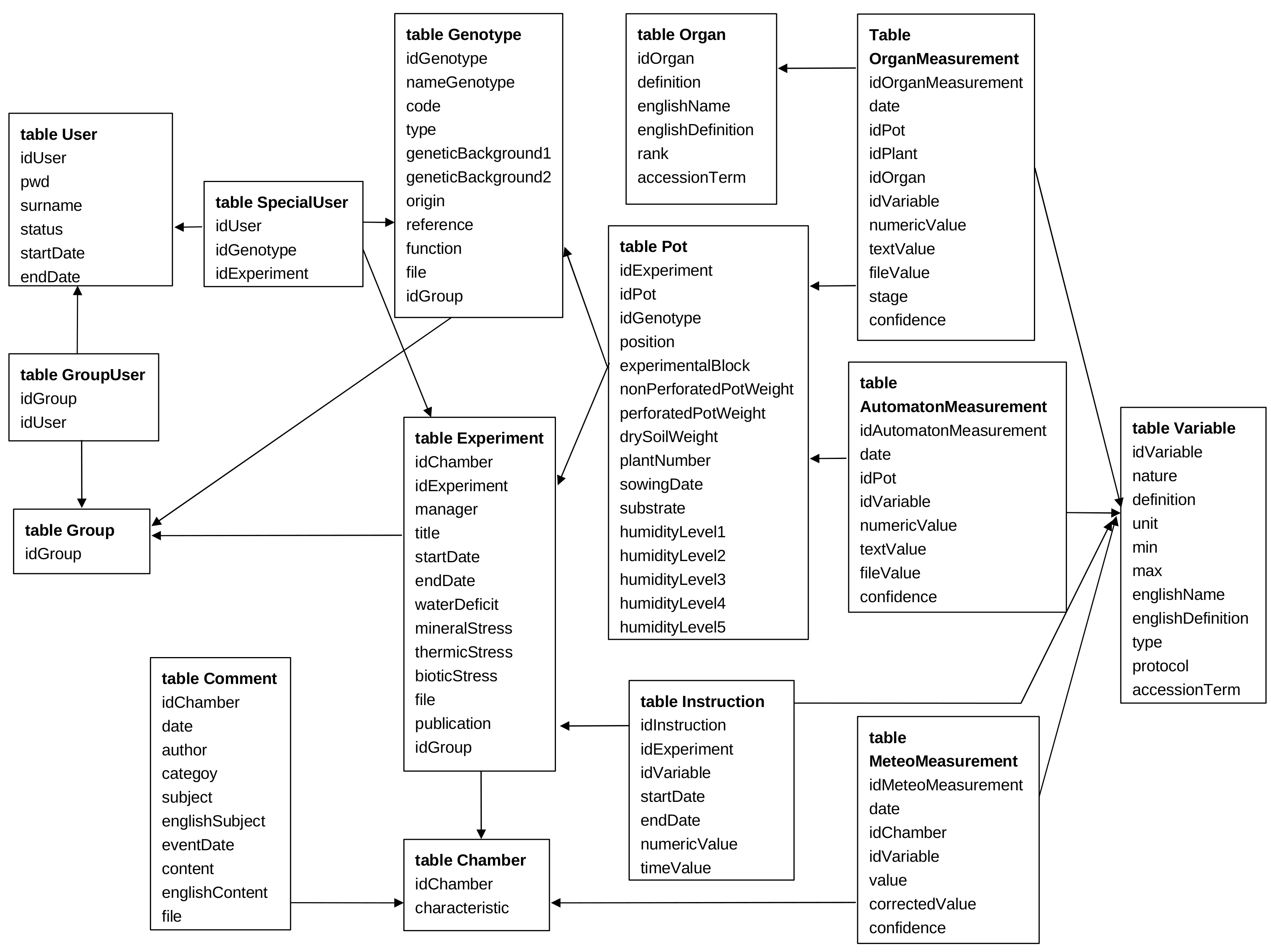

Supplement: Additional file 2 — Description of the physical data model of the PHENOPSIS DB database. Four tables allow the management of user rights (Group, User, SpecialUser and GroupUser tables). They provide authorisation on data access and data insertion and restrict the access to specific experiments and/or genotypes listed with their characteristics in the Experiment and Genotype tables respectively. The growth chamber in which a particular experiment is performed, the characteristics of the pots in this experiment and the environmental instructions provided by experimenters are listed in the Chamber, Pot and Instruction tables respectively. Five other tables are related to the studied variables and the parts of the plants they are measured on. All studied variables are defined in the Variable table and the plant parts on which they are measured are defined in the Organ table. Micro-meteorological data are stored in the MeteoMeasurement table. Plant watering data and names and filename of the images collected by the automatons are stored in the AutomatonMeasurement table. Offline phenotypic data are stored in the OrganMeasurement table, as well as file names of plant images taken by experimenters. A last table named Comment allows the storage of all events and remarks associated with an experiment. Additional supplementary material is available on the PHENOPSIS DB Web interface: http://bioweb.supagro.inra.fr/phenopsis/. [file 1471-2229-11-77-S2.PNG]
